# Effect of Ramadan fasting on disease activity in patients with rheumatoid arthritis presenting in tertiary care hospital

**DOI:** 10.12669/pjms.36.5.2099

**Published:** 2020

**Authors:** Samara Siddique, Yaser Imran, Muhammad Naeem Afzal, Uzma Malik

**Affiliations:** 1 Dr. Samara Siddique, FCPS (Medicine), Trainee Rheumatology, Department of Medicine (EMW), King Edward Medical University, Lahore, Pakistan; 2 Dr. Yaser Imran, FCPS (Medicine), FCPS (Rheum), Assistant Professor of Medicine, Department of Medicine (EMW), King Edward Medical University, Lahore, Pakistan; 3 Dr. Muhammad Naeem Afzal, FCPS (Medicine), Associate Professor of Medicine, Department of Medicine (EMW), King Edward Medical University, Lahore, Pakistan; 4 Dr. Uzma Malik, FCPS (Medicine), FCPS (Endo), Assistant Professor of Medicine, Department of Medicine (EMW), King Edward Medical University, Lahore, Pakistan

**Keywords:** Disease activity score, Rheumatoid arthritis, Ramadan fasting

## Abstract

**Background and objective:**

Rheumatoid Arthritis (RA) is a common inflammatory disorder affected by various factors, including fasting. The objective of the study was to establish the effect of Ramadan fasting on DAS 28 in Rheumatoid Arthritis patients.

**Methods:**

In this observational cohort study done in department of rheumatology, Mayo hospital, Lahore, between May 2019 to July 2019, 240 patients were divided in fasting (n=120) and non-fasting cohort (n=120) based on their own choice. Mean DAS-28 scores before and after Ramadan was compared in both cohorts with appropriate statistical analyses.

**Results:**

Two hundred forty participants, (74 males, 166 females), were recruited. Baseline DAS of fasting group was significantly low (4.35±0.9) as compared to non-fasting group (5.07±0.91). Paired t-test showed statistically significant improvement in fasting and non-fasting groups in total and in both genders (p=0.000). Mean improvement in DAS was numerically greater and statistically significant (p=0.000) in non-fasting group (1.08±0.62) as compared to fasting ones (0.86±0.61). Post-Ramadan DAS was, however, significantly low in fasting group (3.49±0.9) versus non-fasting group (3.98±1.0) (p=0.000).

**CONCLUSION:**

DAS 28 score decreased in both non-fasting as well as fasting patients of RA during the month of Ramadan. RA patients with moderate disease activity, who want to keep fast, can be allowed to do so without any fear of disease worsening.

## INTRODUCTION

Rheumatoid arthritis is one of the most prevalent chronic inflammatory arthritic conditions with annual incidence of 0.5-1%, with more predilection for urban population. Prevalence of Rheumatoid arthritis is increasing in Pakistan. It is 26.9% as reported in 2015.^1^ Multiple genetic and environmental interactions play their role in pathophysiological development of this disease. The disease itself has many associations with co morbidities like dyslipidemias which greatly increases the suffering and worsens disease prognosis.^2^ Immune regulator cells like T-helper 1, T-helper 17, and B cells, along with inflammatory cytokines like TNF-alpha and IL-6, are important in mounting and maintaining inflammation at synovium of joints^1^. While different disease modifying anti-rheumatic drugs like methotrexate, sulfasalazine, and leflunamide, along with steroids are first line drugs for the treatment of rheumatoid arthritis, biologic treatment against TNF-alpha, IL-6, and Cd20 are also gaining expanded role in treatment.^1^,^2^ Diet is also believed to influence the disease activity, with unhealthy diet worsening the disease and Mediterranean diet ameliorating the disease activity.^3^,^4^ Hafstrom et al. described in 1988 that fasting decreased the production of neutrophilic cytotaxins and lysozymes, as well as reduced production of leukotriene B4 from neutrophils of rheumatoid arthritis patients. Fasting also improved duration of morning stiffness and ESR in these patients.^5^ Fasting and fasting mimicking diets have been suggested to improve the disease activity in autoimmune diseases including rheumatoid arthritis, by affecting the survival and functions of lymphocytes and lowering the concentration of inflammatory cytokines like TNF-alpha and IL-6, thus favorably altering the immune functions.^6^

Fasting in Ramadan is one of five compulsory pillars in Islam, observed daily from day dawn to sunset for 29 or 30 days in 9^th^ lunar month of Ramadan.^7^ Every Muslim wants to observe fast, but medical advice on some diseases, including rheumatoid arthritis, is not clear about fasting whether disease activity will improve, remain static or worsen with month long daily fasting during Ramadan. There are different observations on effect of fasting during Ramadan. Some small scale studies have documented beneficial effects of fasting in patients with rheumatoid arthritis.^8^,^9^ Another study shows insignificant improvement^10^, but large scale evidence is missing. So we conducted this observational cohort study to see the effects of fasting in patients with rheumatoid arthritis who are stable with treatment and compliant with medications.

## METHODS

This prospective cohort study was done in department of rheumatology in a large tertiary care hospital. A non-probability convenient sampling method was used to include the cases. Patients already diagnosed as having Rheumatoid Arthritis between 16 to 60 years of age who were consistently taking treatment were included in the study. Those patients with RA who were planning to fast throughout the month of Ramadan were included in Fasting group while others were kept in non-fasting group. Fasting was totally patients’ own choice and we did not enforce it. One hundred twenty patients were included in each group. RA patients who were pregnant, those who were lactating, with severe comorbidities and patients who were in induction phase (with biologic agents) of their treatment were not included in the study. Information like demographic factors as age, gender was also noted while collecting data. Both groups were assessed clinically for disease activity of RA using DAS 28 SCORE (disease activity score) using online calculator, both at the start of study and after Ramadan. All information was taken on a specially designed proforma after taking written and verbal informed consent from the patients.

Data was entered in SPSS 26. Quantitative variables like age were presented as mean+/- SD. Qualitative variables like gender were presented as frequency and percentages. Comparison of DAS before and after Ramadan was done by applying paired t-test in each group (fasting and non-fasting). Mean difference in DAS (before and after Ramadan) between both groups was compared by independent sample t-test. P value < 0.05 was taken as significant.

### Ethical approval

This study was approved by ethical committee of King Edward Medical University (KEMU), Lahore, on August 6,2019 via order no. 1000/RC/KEMU.

## RESULTS

A total of 240 participants, 74 males and 166 females, were included in study. Among females 90 (54.2%) opted for fasting as compared to males (30, 40.5%), but difference was not statistically significant. Mean age of study population was 37 years: that of fasting group was 39, and of non-fasting group was 36 years. Baseline DAS of fasting group was significantly low (4.35±0.9) as compared to non-fasting group (5.07±0.91). Paired t-test showed statistically significant improvement in both fasting and non-fasting groups and in both genders. Mean improvement in DAS was numerically greater and statistically significant in non-fasting group (1.08±0.62) as compared to fasting ones (0.86±0.61). Post-Ramadan DAS was, however, significantly low in fasting group (3.49±0.9) versus non-fasting group (3.98±1.0). Differential effect of fasting on mean DAS score was less pronounced and statistically insignificant in females. Age group stratified analysis was not done due to low numbers of subjects in some cells.

**Table-I:** Comparison of Pre and Post Ramadan das score in Fasting and Non-Fasting Groups.

	Total			Males (n=74)			Females (n=166)		

	Fasting (n=120)	Non-fasting (n=120)	P-value	Fasting (n= 30)	Non-fasting (n= 44)	P-value	Fasting (n= 90)	Non-fasting (n= 76)	P-value
Age group (16-30/31-50/>50)	23/89/08	50/69/01	0.000	9/17/04	19/25/0	0.037	14/72/04	31/44/01	0.000
Pre-Ramadan DAS	4.35±0.9 (0.08)	5.07±0.91 (0.083)	0.000	4.27±0.81 (0.15)	5.23±0.97 (0.15)	0.000	4.38±0.93 (0.098)	4.98±0.87 (0.099)	0.000
Post-Ramadan DAS	3.49±0.90 (0.08)	3.98±1.0 (0.09)	0.000	3.64±0.84 (0.15)	4.15±1.01 (0.15)	0.024	3.44±0.92 (0.097)	3.89±0.99 (0.11)	0.003
Mean Difference in DAS	0.86±0.61 (0.055)	1.08±0.62 (0.056)	0.005	0.63±0.69 (0.12)	1.07±0.59 (0.09)	0.004	0.93±0.57 (0.06)	1.09±0.64 (0.07)	0.10
(Pre- vs Post-Ramadan) Paired t-test	0.000	0.000	--	0.000	0.000	--	0.000	0.000	--

Values in each box (except p-values) represent mean±SD (SEM).

## DISCUSSION

Our study gives evidence about safety of Ramadan fasting in patients with Rheumatoid arthritis which is a very prevalent disease in Pakistan as shown by Syed Mahfooz Alam in a study done in Karachi which shows huge burden of RA at rheumatology clinic.^11^ Our study was a good sample size study. It verifies the results of previous studies about safety of Ramadan fasting in arthritis patients^12^, showing statistically significant improvement in disease activity score in patients who fasted in the month of Ramadan. Fasting improved DAS score in both genders.

New thing reported in our study was that non-fasting group showed numerically greater improvement as compared to fasting group. This trend was present in total as well as in males. However, although numerically similar, yet mean difference or improvement in DAS was less pronounced in females with and without fasting. It was contrary to that reported in previous studies where fasting group had greater reduction in DAS.^8^-^10^ Disease duration and disease activity might have an important modification role in effect of fasting. In study from Bahrain, fasting group had milder disease at baseline, while non-fasting group had moderate disease with longer duration. Fasting people showed significant improvement in global patient assessment. Though non-fasting group didn’t show significant global improvement, yet number of painful joints decreased in them also. In our study, both groups had moderate disease activity, though non-fasting group had significantly higher DAS than fasting ones. Further studies are needed to see the effect of fasting in rheumatoid arthritis patients with reference to functional class, and disease duration.^8^ Another plausible mechanism in this difference can be the effect of dietary changes customary during Ramadan in our population. Though it needs elaboration, in our study, some dietary factors might have precluded or balanced the positive effect in fasting group. Sugary drinks and sweets are known to have negative impact on RA.^13^ In a study from Malaysia, dietary habits were also explored during fasting. Fasting people decreased their use of rice and high calorie food that may be an important contributory factor causing marked improvement in patients who fasted during Ramadan.^10^ Certain dietary interventions, like fruits, whole grains and cereals, legumes, yogurt, essential fatty acids, olive oil and fish oil, and certain herbs improve the disease activity in patients with rheumatoid arthritis.^14^ Dietary antioxidants like omega 3 fatty acids and essential trace elements^15^, as well as use of Mediterranean diet^16^ are associated with reduced severity of inflammatory arthritis. Michelson et al. observed clinical improvement in fasting group as compared to non-fasting.^17^ Similarly according to Dorra B Nessib, in the light of a study done in Feb 2020, Fasting may be a feasible option to bring about fast improvement activity of Rheumatoid Arthritis.^18^

### Limitation of the study

Because month of Ramadan brings important modifications in macronutrient composition of person’s diet, inability to monitor potential dietary changes was an important limitation in our study. We did not collect the diet data in our fasting patients. As high fat diet is known to worsen the inflammatory arthritis in mice^19^, it will be interesting to see the effect of excessive use of fried foods, commonly taken in Iftar in Pakistan, on disease activity in rheumatoid arthritis. We suggest further studies on these lines. A recently published review documents that Ramadan fasting has mild and transient effects on immune system.^12^ A large clinical trial is going on in Germany by Andreas et al. to see the effect of therapeutic fasting and vegan diet on the RA disease activity.^20^ We suggest similar trial with large sample size to be conducted in month of ramazan on patients of rheumatoid arthritis to see if these beneficial effects of fasting persist or diminish over time.

## CONCLUSION

Fasting is safe in patients with rheumatoid arthritis who are in moderate disease activity and are stable on treatment. Further studies are needed to explore the effect of diet and disease duration on beneficial effects of fasting in these patients.

### Author`s Contributions

**SS** conceived, designed and did statistical analysis, editing of manuscript, and responsible for integrity of research.

**UM** did data collection and write manuscript.

**NA and YI** did review and final approval of manuscript.

## References

[1] Naqvi AA, Hassali MA, Aftab MT (2019). Epidemiology of Rheumatoid Arthritis, clinical aspects and socioeconomic determinants in Pakistani patients:A systematic review and meta-analysis. J Pak Med Assoc.

[2] Erum U, Ahsan T, Khowaja D (2017). Lipid abnormalities in patients with Rheumatoid Arthritis. Pak J Med Sci.

[3] Tedeschi SK, Costenbader KH (2016). Is there a role for diet in the therapy of rheumatoid arthritis?. Curr Rheumatol Rep.

[4] Forsyth C, Kouvari M, D'Cunha NM, Georgousopoulou EN, Panagiotakos DB, Mellor DD (2018). The effects of the Mediterranean diet on rheumatoid arthritis prevention and treatment:a systematic review of human prospective studies. Rheumatol Inter.

[5] Hafstrom I, Ringertz B, Gyllenhammar H, Palmblad J, Hams-Ringdhal M (1988). Effects of fasting on disease activity, neutrophil function, fatty acid composition, and leukotriene biosynthesis in patients with rheumatoid arthritis. Arthritis Rheum.

[6] Choi IY, Lee C, Longo VD (2017). Nutrition and fasting mimicking diets in the prevention and treatment of autoimmune diseases and immunosenescence. Mol Cell Endocrinol.

[7] Fasting during Ramadan [Internet] (2019). En.wikipedia.org.

[8] Al-Dubeikil KY, Abdul-Lateef WK (2003). Ramadan fasting and rheumatoid arthritis. Bahrain Med Bull.

[9] Munshi IY, Iqbal M, Rafique H, Ahmad Z, Rashid S (2008). Role of diet in disease activity of arthritis:a questionnaire based survey. Pak J Nutr.

[10] Said MSM, Vin SX, Azhar NA, Jeans Y, Abdullah MNH, Rajalingham S (2013). The effects of the Ramadan month of fasting on disease activity in patients with rheumatoid arthritis. Turk J Rheumatol.

[11] Alam SM, Kidwai AA, Jafri SR, Qureshi BM, Sami A, Qureshi HH (2011). Epidemiology of rheumatoid arthritis in a tertiary care unit, Karachi, Pakistan. J Pak Med Assoc.

[12] Adawi M, Watad A, Brown S, Aazza K, Aazza H, Zouhir M, Sharif K (2017). Ramadan fasting exerts immunomodulatory effects:Insights from a Systematic Review. Front. Immunol.

[13] Skoczynska M, Swierkot J (2018). The role of diet in rheumatoid arthritis. Reumatologia.

[14] Khanna S, Jaiswal KS, Gupta B (2017). Managing rheumatoid arthritis with dietary interventions. Front Nutr.

[15] Millsop IW, Bhatia BK, Debbaneh M, Koo J, Liao W (2014). Diet and psoriasis. Part III:role of nutritional supplements. J Am Acad Dermatol.

[16] Barrea L, Balato N, Di Somma C, Macchia PE, Napolitano M, Savanelli MC, Esposito K, Colao A, Savastano S (2015). Nutrition and psoriasis:is there any association between the severity of the disease and adherence to the Mediterranean diet?. J Transl Med.

[17] Michelson A, Riegert M, Ludtke R, Backer M, Langhorst J, Schwickert M (2005). Mediterranean diet or extended fasting's influence on changing the intestinal microflora, immunoglobulin A secretion and clinical outcome in patients with rheumatoid arthritis and fibromyalgia :an observational study. BMC Complement Altern Med.

[18] Nessib DB, Maatallah K, Ferjani H, Kaffel D, Hamdi W (2020). Impact of Ramadan diurnal intermittent fasting on rheumatic diseases. Clin Rheum.

[19] Suzuki M, Tanaka K, Yoshida H, Yogo K, Matsumoto Y (2017). Obesity does not diminish the efficacy of IL-6 signalling blockade in mice with collagen-induced arthritis. Clin Exp Rheumatol.

[20] Michalsen A Effectiveness of Therapeutic Fasting and Specific diet in patients with Rheumatoid Arthritis a Randomized controlled trial. Started on March 2019 to be completed on July 2020. ClinicalTrials.

